# Signals of energy availability in sleep: consequences of a fat-based metabolism

**DOI:** 10.3389/fnut.2024.1397185

**Published:** 2024-08-29

**Authors:** L. Amber O'Hearn

**Affiliations:** Independent Researcher, Boulder, CO, United States

**Keywords:** adenosine, AMPK, ketogenic diets, metabolism, ROS, sleep, satiety, orexin

## Abstract

Humans can flexibly switch between two primary metabolic modes, usually distinguished by whether substrate supply from glucose can meet energy demands or not. However, it is often overlooked that when glucose use is limited, the remainder of energy needs may still be met more or less effectively with fat and ketone bodies. Hence a fat-based metabolism marked by ketosis is often conflated with starvation and contexts of inadequate energy (including at the cellular level), even when energy itself is in ample supply. Sleep and satiation are regulated by common pathways reflecting energy metabolism. A conceptual analysis that distinguishes signals of inadequate energy in a glucose-dominant metabolism from signals of a fat-based metabolism that may well be energy sufficient allows a reexamination of experimental results in the study of sleep that may shed light on species differences and explain why ketogenic diets have beneficial effects simultaneously in the brain and the periphery. It may also help to distinguish clinically when a failure of a ketogenic diet to resolve symptoms is due to inadequate energy rather than the metabolic state itself.

## 1 Introduction

Ketogenic diets (KDs) result in a metabolic state distinct from that of non-ketogenic diets: regardless of caloric intake or exact composition, by definition a diet is ketogenic when it leads to a fat-based metabolism marked by sustained ketosis (1). The terms “fat-based metabolism” and “glucose-based metabolism” refer to the primary metabolic fuel in use, which can be measured by respiratory quotient (2). The process of shifting from a glucose-based metabolism to a fat-based one is referred to as “keto-adaptation” (3). The full complement of physiologic and metabolic differences between these states are many and still being elucidated; those most pertinent to energy signaling will be discussed below.

Although some studies and applications of KDs entail hypocaloric energy supply or even starvation, this attribute is not necessary for sustained ketosis in humans, which can be achieved in eucaloric or even hypercaloric conditions provided carbohydrate intake, and to a lesser extent protein intake, are restricted (4). The terms hypo-, hyper-, and eucaloric have an implicit assumption we would clarify, however. Typically when using these terms, experimenters establish a baseline level of intake per individual over a period of weight stability, and then assume for simplicity that this value is a fixed one for the individual. This is, of course, an oversimplification, because of metabolic adaptations, and can lead to absurdities if not phrased carefully.

For example, in a recent study of low carbohydrate, high fat diets (LCHFD) in mice (5), mice given an *ad libitum* obesogenic diet ate more and gained more weight than those on a standard control diet. Hence they were deemed “hypercaloric”. However, some of them were then switched to an *ad libitum* LCHFD. Those mice continued to eat the same level of calories, but ceased to gain weight and reversed pathological symptoms. Although they were still eating food with caloric value roughly equal to the amount that was previously hypercaloric, they were technically no longer hypercaloric, but rather eucaloric at a higher caloric value than before. Critically, these isocaloric conditions both occurred *ad libitum*. That is, depending on the dietary composition, the mice ate to the point of extensive weight gain and metabolic disease in one case, but to weight stability and improved health in another.

This distinction is key for discussing satiation, since physiologically driven satiation can be determined, ultimately, only by observation of *ad libitum* intake. This means that satiation can occur independently of caloric balance, and has important implications for the success of dietary interventions. There are competing models for how food intake is regulated. The energostatic model for the control of food intake places the parameter of regulation on the cellular production of energy. As we will discuss, this model has been used to explain observations in obesity (6, 7) and further to explain connections between metabolism and sleep (8, 9). While a hypothesis directly linking satiation signals to sleep has been carefully described by Nicolaidis (10) (detailed below), what has been less explored before is the potential function of that connection. In this conceptual analysis, we attempt to show how sleep regulation by energy signals may fit into a functional theory of sleep.

Because low glucose availability implies low energy availability in the context of a glucose-based metabolism, but not in that of a fat-based metabolism, the biochemical signals corresponding to low glucose are sometimes mistaken for signals of inadequate energy despite the context of an energy adequate fat-based metabolism. The first aim of this article is therefore to describe the often overlooked differences between hypocaloric diets and eucaloric KDs, and to illustrate this with effects on sleep. Further, the model is used to reframe questions about the differential effects of sleep restriction and total sleep deprivation in human and non-human animal studies.

## 2 Ketosis and the “metabolic switch”

The phrase “metabolic switching” has been used to describe “the body's preferential shift from utilization of glucose from glycogenolysis to fatty acids and fatty acid-derived ketones” during fasting as a specialized mode that's beneficial to periodically turn on (11). Likewise, “the ‘glucose switch' profile” is a description of a hysteresis-like mechanism in which hypoglycemia induces a more lipid-oxidation dependent state by reducing expression of genes that stimulate glucose use in mitochondria (12). This is argued to be beneficial for reducing or reversing the burden of diseases of aging if induced often enough to significantly reduce the lifetime exposure to glucose metabolism. However, switches by nature have multiple positions, and we might equally call a transition from a ketogenic metabolism to glucose utilization a “metabolic switch”. That is, while the authors above portray a fat-based metabolism as a switch away from an implicit default, both modes are arguably valid defaults, as hysteresis works in both directions.

One reason that a glucose-based metabolism has been considered a default, is that much of what we know about the metabolic state of ketosis comes from studies in fasted humans or other animals, which makes fat-metabolism appear to be by necessity transient. While the basic biochemical profile is the same whether or not fat is being eaten, ketosis depends on a combination of low available glucose and glucose production substrate on the one hand, and high available fat on the other. In contrast to most other animals, humans have a high base level of body fat that perpetuates a ketogenic metabolism longer under starvation conditions than would otherwise occur before phase III starvation (see Box 1), characterized by higher rates of protein catabolism, sets in O'Hearn et al. (13). Nonetheless, many of the most informative current studies and reviews focus on the fasted state or otherwise calorically restricted ketogenic diets (KDs). An oft acknowledged drawback to this approach is that keto-adaptation, the full transition from glucose-based into fat-based metabolism, typically takes 2–5 days, and so conclusions drawn after only a few days of intervention may not fully characterize a ketogenic metabolism. A second disadvantage is that observations may result from low energy availability that would differ under fully fed ketogenic states.

Box 1 **Phases of starvation.**

The process of starvation has been divided into phases based on the primary fuel substrate: as repositories of each fuel type run out, metabolism shifts to catabolize the next. Assuming we start with ample glycogen stores, animals normally go through three phases: phase I, corresponding to a glucose-based metabolism; phase II, corresponding to a fat-based metabolism; and phase III, which is also glucose-based, but for which the primary source of fuel is protein from muscle catabolism.

This categorization based on primary substrate is useful even when there is no starvation. To avoid further conflation of energy inadequate and energy adequate very-low-carbohydrate conditions, we will sometimes use the hybrid terms *phase I/II/III metabolism*, which are agnostic about the whether the source is endogenous or exogenous, and have no implication of malnourishment, but still recognize the substrate type in use.

These distinctions highlight the lack of specificity of “fasting-mimicking” as a characterization of KDs.

### 2.1 Energy signals characterizing a fat-based metabolism

The biochemical profile associated with a fat-based metabolism includes, among many other differences relative to a glucose-based metabolism, elevated ketone bodies, adenosine, orexin, AMP-activated protein kinase (AMPK), and homeostatic responses to mitochondrial reactive oxygen species (ROS), including uncoupling proteins. Each of these participates in energy partitioning and signaling.

Ketone bodies have been described in different ways based on origin and function. For example, from an origin perspective, they've been called “byproducts of fat metabolism” [e.g., (14, 15)]. From a functional perspective, they are often though of as an “alternative fuel” to glucose for the brain [e.g., (16, 17)]. While these are accurate descriptions, it is more neutral to describe them as a *transport form of fat* able to cross the blood-brain barrier such that it can be used as an energy substrate *complementarily* to glucose. From a signaling perspective, ketone bodies inhibit muscle catabolism (18). When fat metabolism is high, as in phase II, endogenous glucose production can rely more on its byproducts glycerol and acetone for substrate, and lactate recycling from the Cori cycle (19). They also homeostatically regulate lipolysis (20).

Adenosine, AMKP, and orexin are considered energy sensors. Adenosine accumulates from the breakdown of adenosine triphosphate (ATP), but it is also a source of ATP (21, 22). Hence, high levels can indicate ATP being used faster than it is generated (23). Similarly, AMPK is activated by an increase in the ratio of AMP (adenosine monophosphate) to ATP, where AMP is another result of the utilization of ATP. Orexin is a neuropeptide. As suggested by its name, it is associated with hunger, and it is implicated in appetite, wakefulness, and energy expenditure (24). It is activated by low glucose levels (25–27). Mitochondrial ROS is a byproduct not of ATP use, but its generation, and so it also signals energy availability (see Section 6 below).

### 2.2 Mixed states

These two metabolic states, or modes, have biochemical signatures that generally reflect their complementary functions of building (anabolism) and clearing (catabolism), such that one stimulates and benefits the other. For example, brain-derived neurotrophic factor (BDNF) and fibroblast growth factor 21 (FGF21) have names and structures suggesting they are trophic factors, even though they are more expressed under a fat-based metabolism or catabolic states (28–30). In the case of BDNF, this has mechanistically been attributed to the ketone body beta-hydroxybutyrate, which increases BDNF when administered in various forms and which has also been implicated as the mediator of exercise-induced BDNF (31–35). FGF21 is more controversial in mechanism; there are mixed findings depending on species, tissue, and health of the subject (36–38). But FGF21 is consistently increased in prolonged fasting and protein insufficiency (38, 39) So these substances are stimulated by catabolic states, but functionally appear to be trophic or at least anti-catabolic (40). It has been argued that the growth itself is more stimulated by an anabolic switch, after their upregulation (11).

In general, a glucose-based metabolism reflects a more anabolic, or growth promoting phase, whereas a fat-based metabolism tends to reflect energy release from the breakdown of materials. But it must be emphasized that energy can be released from a baseline glucose-based metabolism (one does not have to be ketogenic to use fat stores, for example) and growth can happen while maintaining chronic ketosis, as evidenced by children on ketogenic diets for epilepsy or babies prior to weaning (41). This is because there can be signals of one mode within the other that don't persist long enough to fully change modes. This hysteresis is characteristic of bistable biochemical systems, which typically result from positive feedback loops or substrate inhibition cycles (42, 43). In particular, this is true of fatty-acid oxidation and glucose metabolism (43, 44). Signatures of one mode in the context of the other mode are normally transient, because if the stimulus persists, metabolism enters the other mode. Middle states are generally avoided, because metabolic regulation of gene expression exhibits hysteresis, such that states tend to attract their full expression and persist (12). Part of this is attributable to the Randle cycle, in which cellular uptake of glucose and fat tend to mutually inhibit each other (45). However, if signals remain mixed, this can be an indication of pathology. With a glucose-centric view of metabolism, this pathological mixed state is often erroneously identified with the signatures of a fat-based metabolism even when in context the signature is appropriate. Examples of this include ketosis conflated with keto-acidosis, or glucose intolerance with pathological insulin resistance (IR). Blagosklonny (46) refers to this latter phenomenon by describing fat metabolism as “benevolent pseudodiabetes”.

### 2.3 Fat oxidation: low energy or high energy?

Metabolic processes associated with fatty acid oxidation (FAO) sometimes have opposing implications for energy status depending on metabolic mode. In the context of a glucose-based metabolism, relying mainly on fat oxidation is a marker of low energy status, because in order for FAO to gain prominence, the contextually primary substrate for energy, glucose, must be reduced. Relatedly, due to the fact that (long chain) fatty acids have longer carbon chains, they require more oxygen for their complete oxidation than glucose does. This is sometimes considered less efficient. When mitochondrial ATP is measured by oxygenation rate, as in Donohoe et al. (47), the spurious conclusion may be drawn that cells are in an energy deprived state, even when there is simultaneous evidence to the contrary, such as increased energy expenditure from voluntary locomotor activity. Similarly, the ratio of the oxidized and reduced forms of nicotinamide adenine dinucleotide, NAD+ and NADH, can be used as an indication of cellular energy-deprivation-derived stress (IBID.), even though it is also consistent with a switch to long chain fatty acids as a primary source of energy, with or without energy scarcity. Yet another example comes from the “energy sensor” AMPK, which increases in response to low energy availability (48). Yet under a fat-based metabolism, higher fat oxidation, ie. higher energy, is also associated with higher AMPK (49). This is further understood by noting that FGF21 stimulates AMPK (50), and FGF21 in turn can be stimulated by a variety of nutritional factors including ethanol, sucrose, and fat (51–54). Because these signals appear mixed, there have been calls for research to discover how KDs can be satiating, despite inducing hunger signals (55).

All of these examples can be reconciled by taking into account the background state. Glucose scarcity in a glucose-based metabolism stimulates signals that, if perpetuated, induce a fat-based metabolism. The reverse is also true. As succinctly put by Mobbs et al. (12), “*a general feature of metabolic regulation is that substrates typically induce the metabolic machinery necessary for their own metabolism*.” But this also implies that high levels of circulating fat will induce features of a fat-based metabolism even when glucose remains high. This creates an important asymmetry, because the presence of glucose prevents full fat adaptation, resulting in discordant signals sometimes indicative of type 2 diabetes.

## 3 Common pathways in satiation and sleep

### 3.1 Measures vs. functions

It turns out there many common pathways in the regulation of hunger and satiation on the one hand, and sleep and waking on the other. To discuss these, it will be helpful to distinguish between a biological state, its measurable markers, and the functions that use these markers as signals (Box 2). In particular, many competing theories of hunger and of sleep differ in their proposed variable of regulation. It matters, for example, whether our theory presupposes that sleep duration is homeostatically regulated, or whether sleep duration is a consequence of some other homeostatically regulated variable. In this section, we will review evidence that the primary regulated variable in both satiety and sleep is energy availability, and that this commonality underlies their overlapping pathways.

Box 2 **Markers and signals.**

A marker of a biochemical process is just a consistently detectable output that thereby reliably indicates the presence or degree of that process. However, any such reliable indication can therefore carry information to other processes, hence becoming a signal. For example, the presence of high levels of ketone bodies in the bloodstream contains the information that protein derived from muscle is less required than under the same low glucose condition with lower fat oxidation as in phase III metabolism (see main text).

### 3.2 Satiation, satiety, and the energostatic model

Satiation is the component of cessation of desire to continue eating attributable to physiological signals– as opposed to desires based on external factors that could supersede attention to such signals, for example, the desire to escape a predator, or the desire to curtail a meal based on belief that reduced eating will improve physical fitness. Satiety is the analogous absence of desire to begin eating again, and thus can be thought of as the persistence of satiation signals across time.

The energostatic (or ischymetric) model of satiety, originally introduced by Booth (56), uniquely acknowledges satiety regulating effects of energy production at the cellular level agnostic of source. For an in-depth explanation of this model [see Friedman (6)]. A key insight of the energostatic model is that of locating the parameter of regulation to *energy production* rather than body fat or energy balance *per se*. Hence, for example, obesity is seen not as a result of dysregulation of the homeostatic control of a fat mass set point, but of adaptation to energy production challenges. Accordingly, sensed energy, not fuel, stores, or other “potential energy” (which may or may not actually become ATP), is the mechanism leading to satiation. An important result of the focus on energy sensing is that it helps elucidate tight, bidirectional links between sleep and energy homeostasis.

### 3.3 Measures and functions of sleep

While there are many measurable aspects of sleep, we will focus on duration for each of the two main sleep stages, rapid eye movement (REM) and non-REM (NREM), and the intensity of slow wave activity (SWA) during the latter. REM is characterized by brain activity strongly resembling that of waking, and it was therefore originally called “paradoxical” sleep by Michel Jouvet in 1959 (57), whereas brain activity is reduced and differently patterned in slow wave sleep (SWS).

The drive to sleep has primarily been associated with SWA, such that its absence creates pressure for sleep that is relieved only by SWA. Increased sleep pressure leads to higher intensity of SWA, rather than longer duration. Sleep is therefore normally considered to be homeostatically regulated via SWA. Although there are at least a dozen published theories describing the relationships between NREM and REM (58), much data supports a dependence of REM on NREM suggesting that REM is at least partially homeostatically regulated by SWA (58–60) However, REM is also subject to increased drive when suppressed independently of NREM. Unlike the case with NREM, it is REM duration that appears to be regulated, such that there is a rebound of increased duration after suppression (61). The mechanisms of REM drive are not known, but there is evidence that BDNF is required to stimulate it (IBID). However, BDNF expression also increases SWA in NREM sleep (62) leaving the possibility of common causes in the regulation of both stages.

While the full complement of the functions of sleep is yet to be elucidated, the basic intuition that sleep is restorative and anabolic is at best incomplete, partly because the functions of REM and NREM evidently differ, and partly because some consequences of sleep are clearly more catabolic than anabolic. On the one hand there is evidence of protein synthesis (63). On the other, for example, during NREM sleep there is clearance of metabolites and toxins (64). There is also extensive synaptic pruning, leading to the synaptic homeostasis hypothesis (65), which proposes that continuous learning during waking periods make the brain too expensive to operate, such that synapses must be regularly pruned to allow learning to continue. Hence there is a “restoration”, but the restoration is not only one of rebuilding lost tissue, but also tearing tissue down.

Benington and Heller (66) first suggested that NREM sleep may function to restore brain energy. They reasoned that the increased release of adenosine synthesized from AMP that is associated with increasing sleep intensity must either reflect compromised metabolic supply or increased metabolic demand. The latter was rejected as unlikely due to the observation that neurons are quiescent during slow wave sleep. While it is true that energy use does thus decrease, it is also notable that the onset of NREM is accompanied by a surge in ATP generation from a variety of substrates including glycogen, lactate, and adenosine accumulated during waking (67). Moreover, wakefulness and low energy supply are strongly linked, as discussed below. This link is so reliable that the following hypothesis was formed.

### 3.4 Nicolaidis' hypothesis of satiation and sleep

Based on multiple lines of experimental evidence, Nicolaidis (10) proposed the following hypothesis: “Whenever a neurosubstance is shown to induce satiety, it may also be somnogenic, and it should also increase the background metabolic rate. Conversely, whenever a neurosubstance is shown to be orexigenic, it should also promote wakefulness and, at the same time, decrease the background metabolic rate.” The hypothesis is motivated by the following observations on energy balance and sleep.

## 4 Energy balance in sleep

### 4.1 Duration relationships

While short sleep is considered a risk factor in obesity (68), many studies have found a direct relationship between weight gain and sleep duration: animal studies have found increases in NREM and in some cases REM proportionate to induced weight gain (but not energy intake *per se*), and decreases during loss (69–72). In humans, it has been noted that anorectics sleep less than non-anorectics, that this reduction is rescued in recovery, and that obese people losing weight also have reductions in sleep (73). This reduction appears to effect mainly REM sleep, suggesting that while SWS is promoted by energy use, REM has a stronger requirement for energy availability. This is supported by the fact that other high REM situations are associated with fat gain, such as in infancy (74) and after high carbohydrate meals (75). It is also part of the basis of the energy allocation model of sleep (76), which posits that REM requires so much energy that it shuts down peripheral energy use, such as thermoregulation, in order to reallocate that energy for its functions while minimally affecting total expenditure.

Sleep duration is also positively related to energy intake of the previous meal (8, 9), but, importantly, this effect was not observed by these authors in fat animals under the initial days of starvation. This suggests that it is not the weight loss itself that causes short sleep, but rather the low availability of energy; animals that are able to provide adequate energy from stores do not have reduced sleep duration.

**Figure 1:**
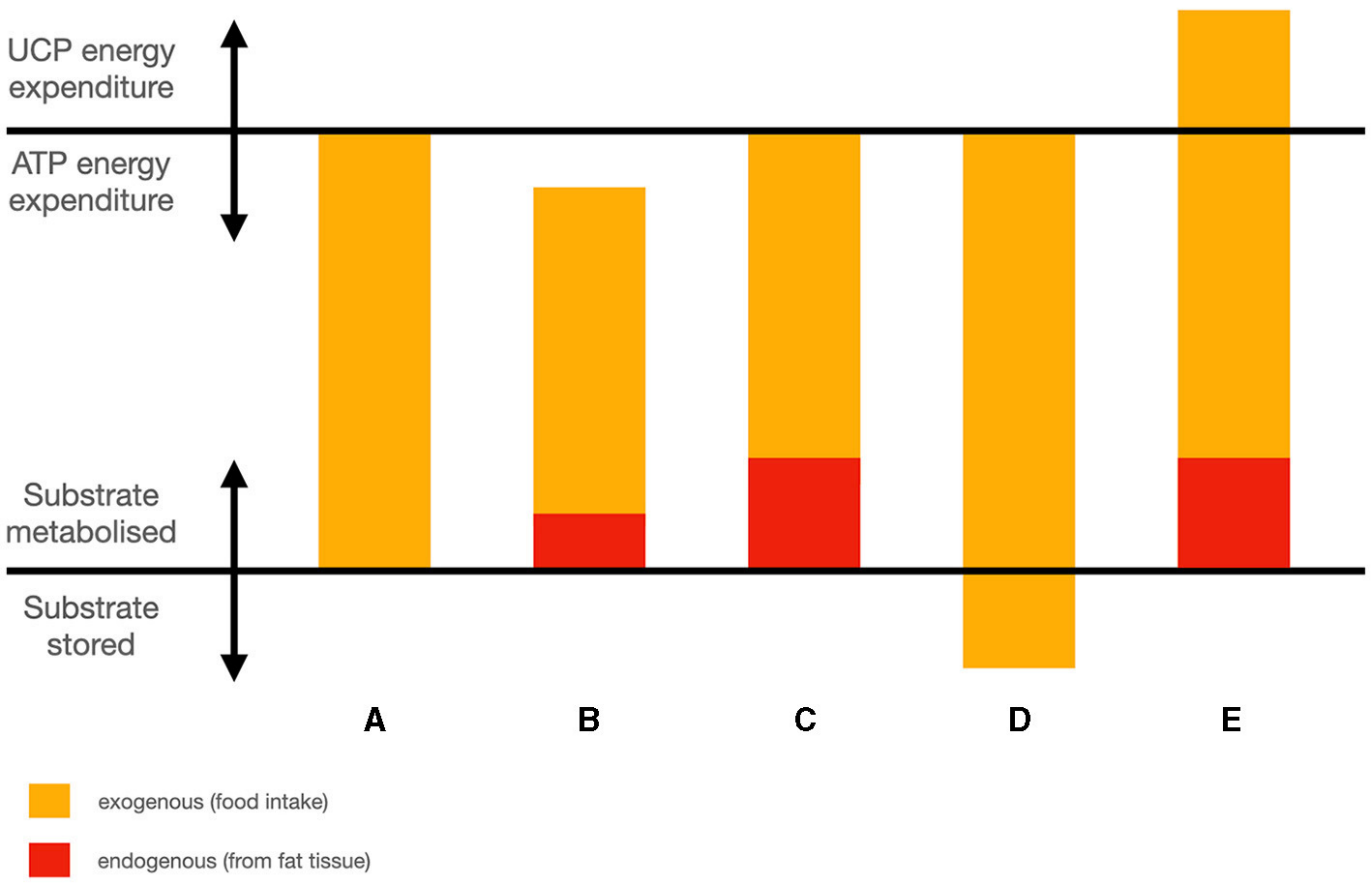
Simplified schematic of fuel partitioning components: **(A)** weight stable; **(B)** weight loss, energy inadequate; **(C)** weight loss, energy adequate; **(D)** higher intake, weight gain; **(E)** higher intake, weight loss.

Along the same lines, a group of 80 overweight humans with chronic short sleep spontaneously ate less and lost weight when subjected to sleep extension from < 6.5 to 8.5 h time in bed (77). As energy expenditure was not changed, the implication is that mild sleep restriction would likewise increase energy intake without increasing energy expenditure. Spontaneous reduction of intake is particularly interesting because it implies that there has been an increase in energy use from body stores contributing to earlier satiation. This contrasts with weight loss from caloric restriction, which may take place in the absence of physiological satiation, suggesting that energy needs are not fully compensated for by fat stores, and total energy is inadequate. Thus, Tasali et al.'s results are consistent with sleep restriction reducing the ability to use glucose via inducing a ‘mixed state’, hence reducing available energy from food which then must be “lost” to fat storage. These concepts are illustrated in Figures 1A–D. Like the case above in which over-fat animals did not lose sleep during the initial days of starvation because using their fat mass made up the difference in energy, in these subjects, sleep extension was associated with continued satiation, even though they ate less. These apparent counter-examples to the general association between weight loss and reduced sleep reveal a refined association by taking into account energy use: weight loss is associated with shorter sleep when it is accompanied by low energy access, but may accompany normal duration of sleep when energy access is adequate.

### 4.2 The effects of a fat-based metabolism on sleep

The effects of fasting and KDs on sleep have been reviewed previously (78). While there have been normalizations of pathological REM duration, the primary effect of a ketogenic metabolism is an increase in SWS. O'Hearn hypothesized that increased brain energy as a result of the KD is responsible for the increased SWA, and that this may be a contributing factor to the cognitive and neurological benefits of KDs. An important observation in that discussion is that orexin signals hunger and wakefulness in the context of a glucose-based metabolism, because it is a marker of low glucose, but in the context of an energy adequate KD, it is high despite satiety and normal sleep duration. Increases in adenosine were also noted (IBID). Accordingly, it was further suggested that the simultaneous increases in adenosine and orexin, which are normally in signaling opposition—adenosine signals satiety and sleep, whereas orexin signals hunger and wakefulness—permit higher levels of adenosine to accumulate with less sleep pressure, which could contribute to the anti-convulsant (79–84) and anti-depressant (85) properties of adenosine without compromising wakefulness.

## 5 Sleep deprivation and metabolism: apparently conflicting data

Sleep restriction (SR) is a form of partial sleep deprivation characterized by reduced sleep duration. SR has often been compared with total sleep deprivation (TSD). For example, Van Dongen et al. (86) compared impairment from accumulated SR to that from TSD. As those authors show, at least in some ways sleep deficit may produce a dose-response effect, with TSD inducing the most extreme rate of accumulation. This approach has been followed by others, for example, Lim et al. (87) compare them for resulting sleepiness and risk taking, Groeger et al. (88) compare them for positive and negative affect changes, and Dennis et al. (89) compare them in resulting compensatory eating. These kinds of similarities and the practical importance of SR as well as sleep fragmentation and circadian desynchrony in modern society has spurred more research comparing these types of sleep disturbances (90).

However, it is important to recognize that there can be qualitative differences between SR and TSD. For example, SR disproportionately affects REM compared to SWA (91–93). This architectural difference implies that SWA takes some priority, a finding with important implications for sleep homeostasis, as well as for the cause of behavioral and physiological impairments under SR (94). For this reason, SR and TSD must be compared with care. Also, because of this cumulative property, predictions of performance based on TSD do not translate with equal accuracy to SR without taking sleep history into account (95).

In the context of studying the potentially causal relationships between sleep deprivation (SD) and obesity, previous authors have observed the puzzling contrast between the negative energy balance in rats induced by TSD, despite the positive correlation between short sleep and obesity in human populations (96, 97). On the one hand, as mentioned above, short sleep is considered a risk factor for obesity. This is based partly on evidence from observational studies, such as from (68, 98, 99). The latter form part of the basis of the oft-cited recommendation on healthy sleep duration: obesity has the lowest prevalence at somewhere between 7 and 8 h per night. Longer sleep is also more correlated with higher weight, but this is reasoned to be due to a common cause, where longer sleep is due to conditions that cause fatigue or other disability leading to lower energy expenditure. On the other hand, the association with short sleep is thought to be a result of glucose intolerance and IR. Short sleep in the acute-term reliably results in higher blood glucose responses to meals (100, 101). Since loss of blood sugar control is an early sign of diabetes which is closely related to obesity, it would stand to reason that repeated exposure to this glucose intolerance adds up to the observed long-term association. Moreover, short sleep increases appetite (99).

However, animal studies on total sleep deprivation (TSD) tell a mostly opposite story. It takes rats about 2–3 weeks to die from TSD (102). Before death, TSD reliably results in reduction in core temperature, elevated energy expenditure, weight loss despite hyperphagia, increased catecholamines, and reduced thyroid hormones (103–105). The elevation in energy expenditure is caused by mitochondrial uncoupling (103) to such a degree that before death, rats are expending more than twice baseline rates (106). In mice, ketone bodies are reportedly elevated in the brain (107) indicating inadequate glucose supply, along with increased AMPK (108) In many respects rodents respond to TSD as if experiencing starvation or adaptation to cold, even though food intake is increased and TSD did not reduce RQ in rat models, possibly simply because of their excessive carbohydrate intake (103) (see Table 1).

**Table 1:** Energy signals in various states.

	**GB-F**	**GB-LG**	**KD-F**	**GB-TSD**	**GB-SR**
KBs	−	−	+	+	−
orexin	−	+	+	+	+
AMPK	−	+	+	+	?
mtROS	+	−	+	+	?
UCPs	−	−	+	+	?

GB-F, glucose-base, fed; GB-LG, glucose-based, low glucose; KD-F, ketogenic diet, fed; GB-TST, glucose-based, total sleep deprivation (in rodents); GB-SR, glucose-based, sleep restricted (humans). “+” indicates elevated relative to a glucose-based metabolism in the post-absorptive phase. − and ? indicates mixed or incomplete research.

These pathways are not confirmed in humans, partly because we cannot expose them to TSD until death. TSD does result in a reduction in core body temperature (76). In studies on sleep *restriction* (SR) as mentioned above, hyperphagia is seen, but energy expenditure doesn't seem to rise enough to match. Energy expenditure does in fact rise, an observation that has been used to support the hypothesis that energy conservation is a function of sleep (63). But this observation is accompanied by the cautionary warning: “The finding that sleep deprivation increases energy expenditure should not be interpreted that sleep deprivation is a safe or effective strategy for weight loss as other studies have shown that chronic sleep deprivation is associated with impaired cognition and weight gain.” (63).

## 6 The role of mitochondrial uncoupling and uncoupling proteins

Mitochondrial uncoupling refers to the process where the transfer of protons across the mitochondrial inner membrane during oxidative phosphorylation is disconnected from ATP synthesis, resulting in the generation of heat instead of storing energy as ATP. However, mitochondrial uncoupling is a subject of multiple controversies. First of all, other than UCP1, it is questionable whether the so-called uncoupling proteins result in uncoupling at all (109, 110). Second, the function of uncoupling when it does occur is disputed. One prominent theory is that mitochondrial uncoupling serves to protect against ROS (see below).

ROS are a normal byproduct of oxidative phosphorylation (111). Although unchecked it can lead to oxidative damage, as a byproduct of a process it is a marker, and hence it signals energy generation. In the hypothalamus, ROS signal satiety (112–115). It's also possible that they signal “satiety” in adipose tissue^1^. ROS have been implicated in IR, the inhibition of glucose uptake via GLUT4 (116–118). While this is normally conceived of as detrimental, because of the connection between IR and diabetes, glucose intolerance at the adipocyte means less adipose expansion. It is a natural cellular signal of “we don't need more energy.”

At the same time, the hypothesis that ROS promote sleep (119) has been supported by experiments showing that SD causes oxidation (120–122), that antioxidants can reduce negative consequences of SD (122, 123) and further, that death by TSD can be prevented via antioxidant intake in drosophilia (124). In one model, this is explained by proposing that one function of sleep is to clear ROS (125). Hence they propose that sleep is regulated by ROS levels. A ROS theory of sleep function is thus consistent with Nicolaidis' prediction: ROS promote sleep and satiety, and ROS are an energy balance signal. If uncoupling is indeed a response to protect against ROS [but see (109)], then the extreme rise in energy expenditure in TSD would also support the ROS clearance function of sleep.

Returning to the criticism that most uncoupling proteins do not uncouple, it is notable that what they do have in common is an upregulation of processes related to lipid metabolism. UCP1 was the first uncoupling protein to be discovered, but is now considered further derived from other UCPs (126). It is associated with mitochondrial uncoupling in brown adipose tissue, which is more prevalent in non-human animals than in humans. UCP2 has a glucose sparing role, reducing the entry of pyruvate into the Krebs cycle and reducing insulin secretion (127). UCP3 enhances fat metabolism (128).

Given that SD promotes uncoupling proteins (129) and KDs promote uncoupling proteins (130–132), and likewise SD and KDs promote glucose intolerance that may be benign in the context of low glucose intake (see Section 2.2), the totality of the evidence suggests that SD is concordant with a fat-based metabolism (Table 1). Hence it is possible that the differences observed between rodent and human responses to SD are attributable to either (a) species differences in uncoupling protein activation, such that humans have hyperphagia without compensatory energy expenditure, (b) an incomplete response to SR, such that ROS first induce IR and only under longer periods of restriction activate uncoupling, or (c) a physiological mismatch between short sleep and glucose intake, such that glucose intake is not concordant with long wake times. The former discordance bears some resemblance to mismatches observed when circadian rhythms are disrupted–eating during times usually reserved for sleep also results in IR. In other words, SD under a glucose-based metabolism creates a metabolic mixed state.

## 7 Discussion

If, as posited by the energostatic model, “energy” is the parameter of regulation for satiety (or anything else), it still must be mediated by a signal or set of signals. Many signals that characterize a fat-based metabolism–ketone bodies, orexin, adenosine, AMPK– are at risk of conflation with signals of energy deficiency. That is, they can be present with or without satiation. Because KDs can result in weight loss, weight stability, or weight gain, we need to look at other signals to determine true energy status.

**Figure 2:**
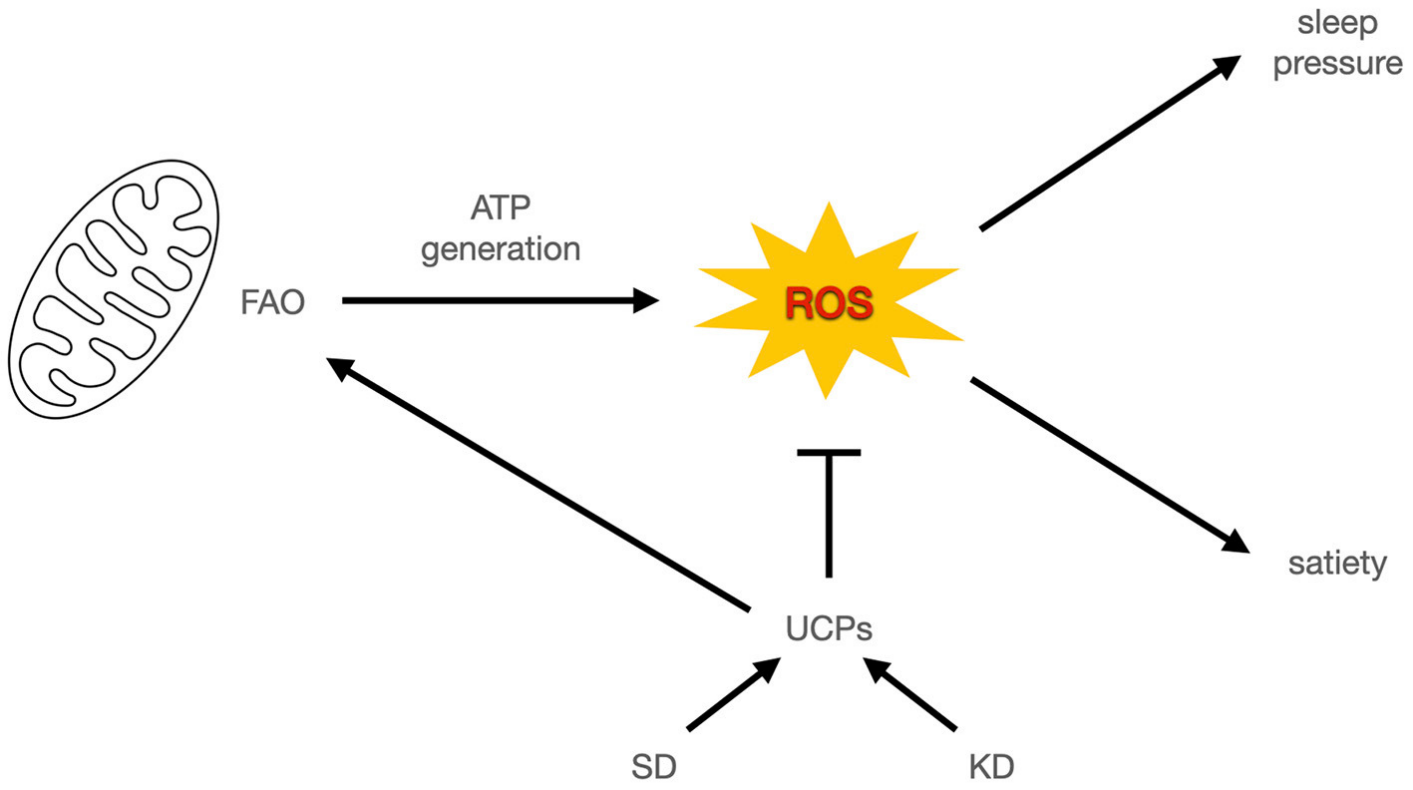
ROS signal energy status in sleep and satiety. In this model, mitochondrial ROS from energy generation signals sleep pressure and satiation. Uncoupling proteins enable more energy expenditure by reducing ROS and decreasing satiation. If this is accompanied by increased energy expenditure it leads to weight loss or weight maintenance at higher caloric intake. Otherwise it leads to weight gain.

Mitochondrial ROS (mtROS) are a candidate exception, because they are signals of satiety in a glucose-based metabolism even though they are also signals of a high energy state in a fat-based metabolism; in the absence of glucose, FAO leads to ROS (as all energy generation does), but also becomes part of a cascade leading to uncoupling which in turn lowers ROS, allowing even more FAO (113). In other words, FAO in the absence of glucose initiates a hysteresis mechanism that helps to bootstrap the fat-based metabolism via increasing ROS tolerance through uncoupling and the resulting increased energy expenditure (Figure 2). Insofar as ROS reduction is an important function of sleep, it could help to functionally explain the previously observed overlap in sleep and satiety signaling. It could also potentially explain why TSD in rodents increases hunger that cannot keep up with energy expenditure, if the uncoupling response to excess ROS burden increases without bound (Figure 1E). It also helps distinguish how a fat-based metabolism can improve sleep quality under fed conditions, even though starvation, which also has a ketogenic metabolic profile, compromises sleep. For a summary comparison of various states discussed and energy signals, see Table 1. A potential area of further investigation is the role of thermoregulation, given the connection of heat generation to uncoupling and the interactions between temperature and sleep quality.

Taken together, these observations support the conceptual framework of energy availability as the target of homeostasis that sleep and satiety regulate. That is, the signals that promote transitions between sleeping and waking on the one hand, and between eating and not eating on the other, are direct consequences of energy availability and use. This framework can then help to explain why KDs have such broad therapeutic value, through the common effect of energy availability in the brain and periphery. In the brain, many neurological and psychiatric disorders have been described as problems of energy access (133). Moreover, sleep problems are frequently comorbidities (134). Insofar as sleep quality reflects adequate energy, the beneficial effects of KDs on sleep may be seen as confirmation of their ability to restore brain energy. At the same time, adequate sleep allows further restorative processes that may directly contribute to therapeutic effects (78). In the periphery, *ad libitum* KDs treat obesity, not by sending signals of reduced energy availability that serve to induce catabolism of fat stores as in caloric restriction regimes, but by sending signals of *increased* energy availability (and hence satiety) when body fat is used. Use of fat for energy during a glucose-based metabolism would be a mixed state, and less likely to send clear satiety signals, with the exception of ROS. Surprisingly, high fat KDs can in some cases treat anorexia (135). While the mechanism for this is unclear, it may be a combination of brain energy effects and restored satiety signaling in response to fat intake.

## Footnotes

^1^I attribute this idea to Peter Dobromylskyj.
